# STarMir: a web server for prediction of microRNA binding sites

**DOI:** 10.1093/nar/gku376

**Published:** 2014-05-06

**Authors:** William Rennie, Chaochun Liu, C. Steven Carmack, Adam Wolenc, Shaveta Kanoria, Jun Lu, Dang Long, Ye Ding

**Affiliations:** 1 Wadsworth Center, New York State Department of Health, Center for Medical Science, 150 New Scotland Avenue, Albany, NY 12208, USA; 2 Department of Genetics and Yale Stem Cell Center, Yale University, New Haven, CT 06520, USA

## Abstract

STarMir web server predicts microRNA (miRNA) binding sites on a target ribonucleic acid (RNA). STarMir is an implementation of logistic prediction models developed with miRNA binding data from crosslinking immunoprecipitation (CLIP) studies (Liu,C., Mallick, B., Long, D., Rennie, W.A., Wolenc, A., Carmack, C.S. and Ding, Y. (2013). CLIP-based prediction of mammalian microRNA binding sites. *Nucleic Acids Res.*, **41(14)**, e138). In both intra-dataset and inter-dataset validations, the models showed major improvements over established algorithms in predictions of both seed and seedless sites. General applicability of the models was indicated by good performance in cross-species validations. The input data for STarMir is processed by the web server to perform prediction of miRNA binding sites, compute comprehensive sequence, thermodynamic and target structure features and a logistic probability as a measure of confidence for each predicted site. For each of seed and seedless sites and for all three regions of a mRNA (3′ UTR, CDS and 5′ UTR), STarMir output includes the computed binding site features, the logistic probability and a publication-quality diagram of the predicted miRNA:target hybrid. The prediction results are available through both an interactive viewer and downloadable text files. As an application module of the Sfold RNA package (http://sfold.wadsworth.org), STarMir is freely available to all at http://sfold.wadsworth.org/starmir.html.

## INTRODUCTION

MicroRNAs (miRNAs) are a class of small endogenous non-coding RNAs (ncRNAs) of ∼22 nucleotides (nts) in length that have been found in plants, animals and viruses. miRNAs are involved in the post-transcriptional regulation of gene expression by binding to target messenger RNAs (mRNAs), leading to translational repression and/or mRNA destabilization (1). They regulate diverse developmental processes, molecular and cellular pathways and are associated with cancer and other human diseases. Prediction and validation of miRNA targets are essential for understanding functions of miRNAs in gene regulation.

Algorithms for the prediction of miRNA:target binding sites are typically based on the seed rule, i.e. the target forms Watson–Crick (WC) pairs with bases two through seven or eight, at the 5′ end of the miRNA (2). However, numerous exceptions to the seed rule have been documented (3–5). In recent years, miRNA binding site data from crosslinking immunoprecipitation (CLIP) studies have become available. These include HITS-CLIP for mouse brain (6), PAR-CLIP for human cell lines (7) and several variants of PAR-CLIP for the same human cell lines (8). CLIP studies generate short target fragments that contain miRNA binding sites. Although the CLIP technique only covers abundantly expressed miRNAs and transcripts in the experimental system, we successfully utilized the high throughput data for developing logistic models to improve binding site predictions for any miRNA:target pair (9). The models adopt the features essential for miRNA binding from a list of comprehensive sequence, thermodynamic and target structure features (10). For model validations, we used five independent CLIP datasets for both inter-dataset validation as well as cross-species validation. Each CLIP dataset yielded at least 10 million binding sites, of which ∼5–15% are positive. For performance evaluation, we calculated true positive rate (TPR = sensitivity), false positive rate (FPR = 1 − specificity) and an overall performance measure: Youden's *J*-statistic (sensitivity + specificity − 1) (11). The models were found to substantially outperform established algorithms. Furthermore, the good performance by the models in cross-species validation suggests that the models can be generally applicable to microRNA binding site prediction for any mammalian species and beyond.

We have implemented the models into the STarMir application module of the Sfold RNA package (12). STarMir web server allows users to submit miRNA and mRNA sequences for prediction of binding sites by the models. For a given pair of miRNA:target mRNA, STarMir first predicts target secondary structures (13). Potential miRNA binding sites are then predicted by the RNAhybrid program for either seed matches or seedless sites with a hybrid stability of −15 kcal/mol or lower (14). For each site, a comprehensive list of sequence, thermodynamic and structure-based features are computed as previously described (9). These features are used by our logistic model with parameters specific for the site type (seed or seedless) and the target region (5′ UTR, CDS or 3′ UTR) to compute a logistic probability as a measure of confidence in the predicted site. In general, a probability of 0.5 indicates a fairly good chance of miRNA binding. A high likelihood of miRNA binding is predicted by a high probability, e.g. 0.75 or higher. In addition, for each site, STarMir also outputs all of the site features along with a diagram of miRNA:target hybrid conformation.

STarMir can be accessed either from the Sfold main page (http://sfold.wadsworth.org), or directly at http://sfold.wadsworth.org/starmir.html. The STarMir web service is freely available to all without a registration or login requirement. In this article, we highlight the main features of the web service. Users are encouraged to consult the online manual by clicking the ‘MANUAL’ button in the STarMir menu line and to examine sample output by clicking the ‘DEMO OUTPUT’ button.

## INPUT

The user needs to input sequence information for one or more miRNAs and a single target mRNA for job processing by the web server. A link is provided for the user to check on the progress of the job and to access the results. Detailed descriptions of the inputs are given below. 

### Model for prediction

The user first selects a prediction model. Currently available models are: a model trained on V-CLIP data for human (*Homo sapiens*) (8), a model trained on HITS-CLIP data for mouse (*Mus musculus*) (6) and a recent model for *Caenorhabditis elegans* based on analysis and modeling of worm ALG-1 CLIP data (15,16).

### Species for prediction

The user next selects the species for prediction. This information will be used if the RefSeq ID is entered for the target mRNA sequence so that the server can utilize pre-stored evolutionary conservation information in the modeling computation. The choice of species has no effect if the mRNA sequence information is entered manually. Furthermore, if ‘other’ is selected, conservation information cannot be used in model prediction. We note that applications of STarMir are not limited to the species with available CLIP data and prediction models, as good performance in cross-species validation suggests that the models can also be applied to other species (9).

### miRNA sequences

**Figure 1.**
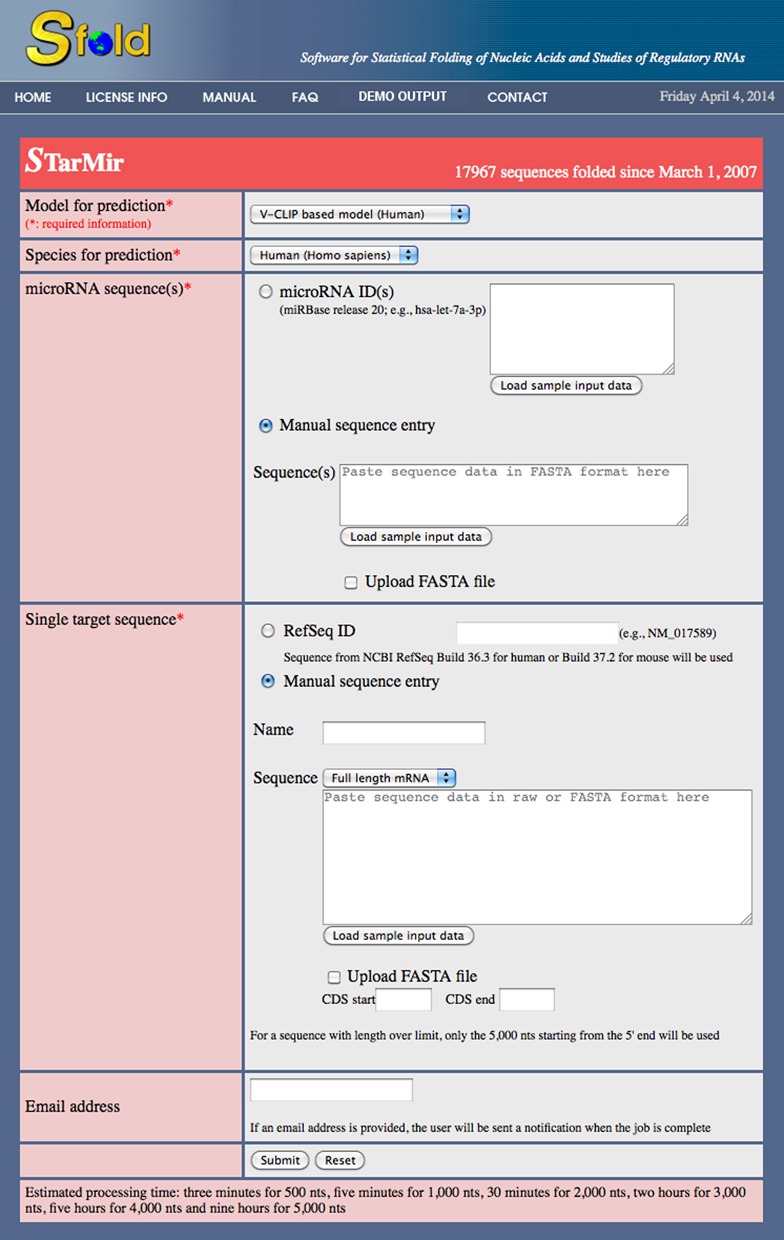
STarMir input page with manual entry option selected for both miRNA and target sequences.

In the default option, the user can enter one or more miRNA IDs, e.g. hsa-let-7a-3p, mmu-mir-128-1, cel-mir-90. For this option, the sequences are retrieved from an internal database built from release 20 of the miRBase (17). Alternatively, one or more miRNA sequences can be pasted into the input box in FASTA format, or uploaded from a FASTA file (Figure 1). There is no limit on the number of miRNA sequences that can be entered. miRNA sequences must be less than 55 nts in length. Any character in the miRNA other than A, T, C, G and U will be removed.

### mRNA sequence

There are three methods that can be used to input the sequence information for the target mRNA. The default method is to enter the RefSeq ID in the input box provided (Figure 1). The sequence will then be retrieved from our database of mRNA sequences. We have stored ∼19 000 sequences from National Center for Biotechnology Information (NCBI) RefSeq Build 36.3 for human and ∼12 000 sequences from Build 37.2 for mouse. If the sequence is specified using the RefSeq ID and is present in our mRNA database, the models will utilize evolutionary conservation information (9,18) for improved predictions. The sequence data can also be provided by selecting the ‘Manual sequence entry’ option to either input the sequence in raw or FASTA format or upload from a FASTA file (Figure 1). When the sequence is loaded from a FASTA format file, the file must contain only one sequence.

Any character in the mRNA sequences other than A, T, C, G and U will be removed. The current web server limit on the length of the mRNA sequence is 5000 nts. Longer sequences will be truncated to 5000 nts starting from the 5′ end

The mRNA region information needs to be provided to the server through the region dropdown box directly above the sequence input box (Figure 1). The user needs to indicate that the sequence entered represents an entire mRNA or a single region (3′ UTR, CDS or 5′ UTR). If the sequence represents the entire mRNA, the nucleotide positions for the start and end of the coding region must be specified in the boxes provided below the input window.

If the sequence is entered via RefSeq ID, the boundaries of the coding region will be retrieved from our mRNA database and binding sites will be reported for all three regions. The name of the sequence for output will be the RefSeq ID. For a manually entered sequence, the user has the option to name the sequence. Provision of an email address is optional. If an email address is entered, the user will receive a notification when the job is complete.

## OUTPUT

The output results are presented to the user through both an interactive viewer and downloadable files.

### Interactive viewer

**Figure 2.**
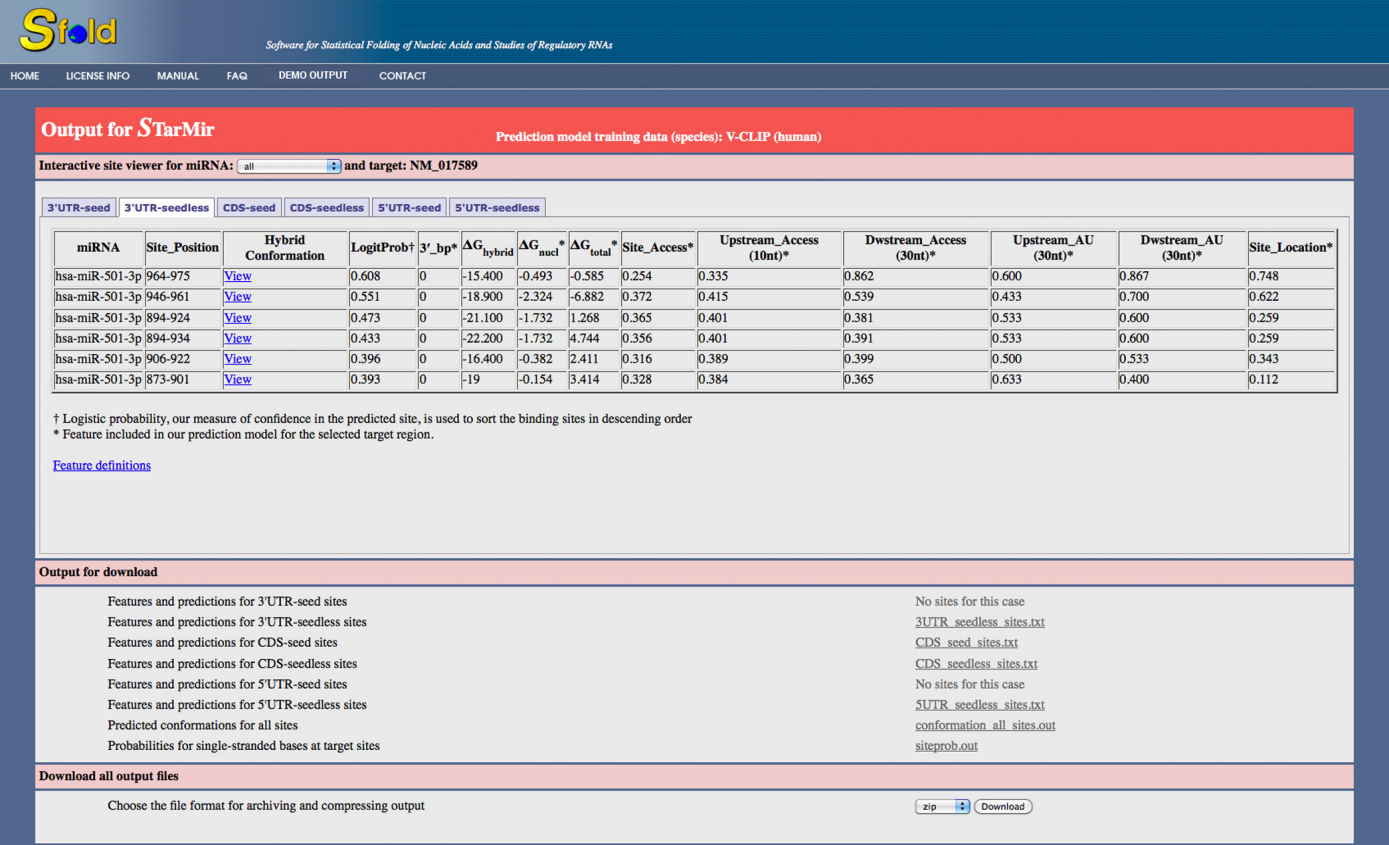
STarMir output page showing the interactive site viewer (with ‘3′ UTR-seedless’ tab selected for display) and the download links for text files.

**Figure 3.**
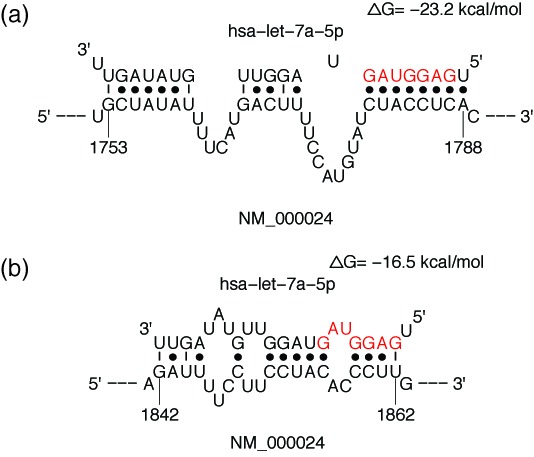
(**a**) Hybrid diagram for a seed site (miRNA seed region (nt 2–8) in red); (**b**) hybrid diagram for a seedless (non-canonical) site.

The results appear in a six-tabbed pane (Figure 2). For ease of use, the results are divided into seed and seedless sites for each of the three target regions (3′ UTR, CDS and 5′ UTR). Within each tab, the results for all or one individual miRNA selected from the dropdown menu in the interactive viewer are presented in a table in descending order of their logistic probabilities. For each binding site, information for comprehensive sequence, thermodynamic and target structure features is provided, along with a logistic probability as a measure of confidence for the site. In addition, a link to a graphic representation of the hybrid conformation and a PDF of the diagram is available for visualization and download (Figure 3). A file for definitions of the features with references is available by clicking the link for ‘Feature definitions’ under the result table.

### Downloadable files

The results can also be downloaded as tab-delimited text files. The text files include all site features calculated by STarMir with the features used in the prediction model marked with an asterisk (*). The prediction models exclude features that were not enriched in CLIP data analysis (9). A text file is available for each of the six categories represented by the tabs. All results can be downloaded as a compressed archive. In addition, there is a file representing a simplified text version of the hybrid conformations for each site and a file presenting the probability that each nucleotide in the site is unpaired (i.e. single-stranded).

### Additional information

STarMir uses output from the Srna module of the Sfold package in the computation of target accessibility measures. The result page contains a dropdown menu providing access to predictions generated by Srna as well as other modules of the Sfold (12).

## CONCLUSION

The STarMir web server enables predictions of miRNA binding sites for any species of interest. The server provides comprehensive site features for both seed and seedless sites to facilitate both experimental and computational investigations. STarMir performs computations including target secondary structure predictions that are time-consuming especially for long target sequences. Thus, it cannot return results instantly as does a database search. A separate database has been under our development for the distribution of pre-computed transcriptome-scale results of select species. STarMir and this database will be complementary tools. While STarMir can make predictions for any miRNA:mRNA pair from any species of interest, the database will allow fast search of pre-computed results for multiple miRNAs and mRNAs.

## CITING THE STarMir WEB SERVER

In research publications, the users of STarMir should cite this article as well as the papers describing the prediction models for miRNA:target interactions (9,10).

## SUPPLEMENTARY DATA

Supplementary Data are available at NAR Online.

Supplementary Data
