# Supplementary material for: STarMir: a web server for prediction of microRNA binding sites
Source: Nucleic Acids Res. 2014 May 6;42(Web Server issue):W114–8. doi: 10.1093/nar/gku376 (PMC4086099; doi:10.1093/nar/gku376)
Supplement: Supplementary Data [file supp_42_W1_W114__index.html]

Supplementary Data 

# STarMir: a web server for prediction of microRNA binding sites

## Supplementary Data

**Files in this Data Supplement:**

- Supplementary Data
